# Mechanobiology in oncology: basic concepts and clinical prospects

**DOI:** 10.3389/fcell.2023.1239749

**Published:** 2023-10-31

**Authors:** Michelle B. Chen, Yousef Javanmardi, Somayeh Shahreza, Bianca Serwinski, Amir Aref, Boris Djordjevic, Emad Moeendarbary

**Affiliations:** ^1^ Department of Biological Engineering, Massachusetts Institute of Technology, Cambridge, MA, United States; ^2^ Department of Mechanical Engineering, University College London, London, United Kingdom; ^3^ 199 Biotechnologies Ltd., London, United Kingdom; ^4^ Northeastern University London, London, United Kingdom; ^5^ Belfer Center for Applied Cancer Science, Dana-Farber Cancer Institute, Harvard Medical School, Boston, MA, United States

**Keywords:** cancer, mechanobiolgy, extracellular matrix (ECM), metastasis (cancer metastasis), invasion, mechanotherapeutics

## Abstract

The interplay between genetic transformations, biochemical communications, and physical interactions is crucial in cancer progression. Metastasis, a leading cause of cancer-related deaths, involves a series of steps, including invasion, intravasation, circulation survival, and extravasation. Mechanical alterations, such as changes in stiffness and morphology, play a significant role in all stages of cancer initiation and dissemination. Accordingly, a better understanding of cancer mechanobiology can help in the development of novel therapeutic strategies. Targeting the physical properties of tumours and their microenvironment presents opportunities for intervention. Advancements in imaging techniques and lab-on-a-chip systems enable personalized investigations of tumor biomechanics and drug screening. Investigation of the interplay between genetic, biochemical, and mechanical factors, which is of crucial importance in cancer progression, offers insights for personalized medicine and innovative treatment strategies.

## Introduction: mechanobiology and cancer

**FIGURE 1:**
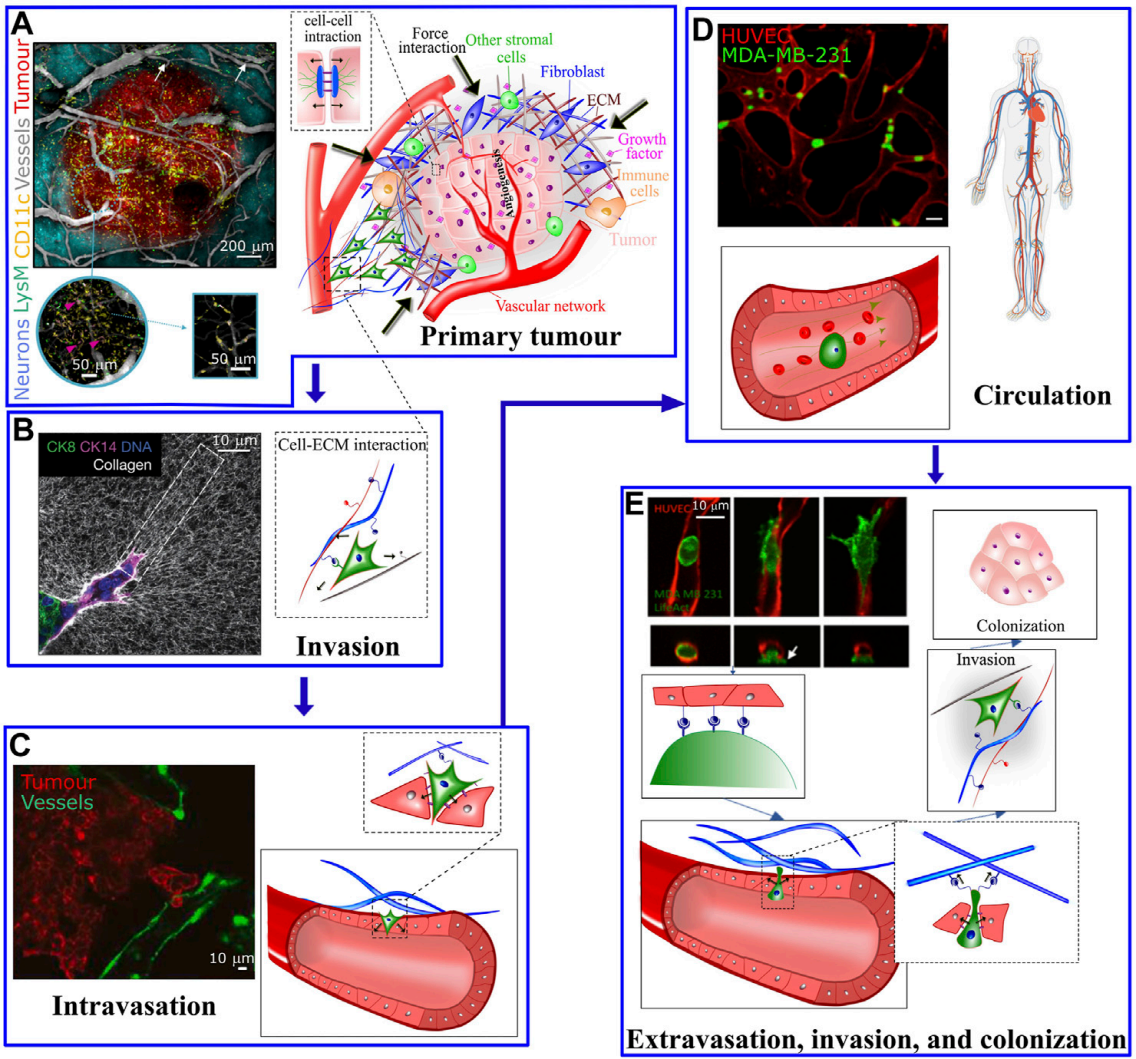
**(A)** The tumour microenvironment at the primary site is very complex; Hypoxic pathways and signaling with other supporting cells, including immune cells, mesenchymal stromal cells and fibroblasts activates endothelial cells to form vascular network in the tumor and surrounding areas. The left panel shows a five-color intravital two-photon image acquired from a bevacizumab-treated triple transgenic mouse. Arrows show the LysM-EGFP^+^ cells present in the vessels. The cells colored in pink refer to double-labelled cells (pink arrows) (CD11c-EYFP+/LysM-EGFP+) inside the tumor [taken from Soubéran et al. (2019)]. **(B)** At the primary tumor site, factors including matrix pore size, density, stiffness, and fiber orientation play a role in modulating the migratory and invasive capabilities of tumor cells. Left panel: Confocal reflection microscopy reveals that invasive organoids align the collagen network to facilitate tumor invasion (taken from Koorman et al. (2022)]. **(C)** Tumor cells cross the endothelial barrier to enter the circulation. **(D)** During circulation tumor cells are exposed to shear stress from the blood flow (left panel in C and D are taken from Agrawal et al. (2022) and Beyer et al. (2021), respectively). **(E)** To exit the circulation, tumor cells pass through the endothelial barrier. The process of transendothelial migration involves significant tumor cell deformations and generation of cellular forces that may activate downstream mechanosensitive pathways. Upper left image is taken from Chen et al. (2017). White arrow depicts actin-rich tumor cell protrusion passing through the endothelium.

Genetic transformations, biochemical communications and physical interactions are interconnected processes involved from the initial steps of tumor formation until the latter phases of cancer metastasis, a major cause of cancer related deaths. Metastatic cascade begins when the primary tumor cells gain aggressive and migratory phenotypes resulting in leaving the primary tumor (Figure 1A), invading the local tissue (Figure 1B) (Craene and Berx, 2013) and transmigrating through the endothelial barrier into the blood or lymphatic microvasculature (a process known as intravasation, Figure 1C) (Van Zijl et al., 2011). The latter steps of disease involve survival of cancer cells in blood circulation (Figure 1D) (Aceto et al., 2015; Reymond et al., 2013) and exit from the vessels at distal tissues (a process known as extravasation) to ultimately invade and colonize in the secondary sites (Figure 1E) (Nguyen et al., 2009). In addition to a number of unique genetic and biochemical factors associated with metastasis (Suresh, 2007; Kumar and Weaver, 2009; Wirtz et al., 2011), irregular mechanical alterations such as structural, morphological and stiffness changes, in both cells and the extracellular environment, play a significant role during all stages of cancer initiation and dissemination. For example, the primary tumor is characterized by biochemically and mechanically altered environment that results from oncogenic mutations and epigenetic changes disrupting key physiological cellular processes such as cell cycle (Spill et al., 2016). Taking advantage of the abnormal mechanical properties of most tumors, palpation has been a conventional diagnostic method to assess the stiffness of tumor within the surrounding soft tissue. The disease stage is linked with tumor stiffness as monitored by *in vivo* MRI elastography or through *ex vivo* atomic force microscopy (Lopez et al., 2011). On the other hand, during tumor growth, the mechanical alterations in tumor environments trigger complex bio-mechanical signalling pathways, which may ultimately enhance the ability of tumor cells to acquire a malignant phenotype. The malignant evolution of cancer cells de-regulates cell-cell and cell-extracellular matrix (ECM) adhesions and cytoskeletal remodeling leading to abnormal tumor cell morphology, enhancement of metabolism (Torrino et al., 2021), and migratory behavior which facilitate invasion at the primary site and ultimately leads to intravasation. Following intravasation, circulating tumor cells (CTCs) must resist the mechanical forces in the bloodstream to survive and reach the secondary organ. Once at the secondary site, tumor cells must exert forces and undergo morphological changes to escape from the vasculature and invade the ECM of the distal organ.

These mechanical interactions during the metastatic journey of tumor cells highlight the importance of biomechanics in cancer dissemination (Kumar and Weaver, 2009; Moeendarbary and Harris, 2014; Malandrino et al., 2018a). Here we describe the crucial interplay between mechanics and biology during cancer initiation and progression together with recent conceptual and technological advances in mechanobiology which have led to a remarkable progress in leveraging cancer biomechanics to develop novel therapeutic strategies.

## Mechanical forces during tumor initiation and growth

Even in a seemingly static tissue, the extracellular microenvironment exerts forces to the cells. Such forces are originated from adjacent cells, the interstitial fluid, or interaction with the ECM, and are sensed via cellular mechanosensitive receptors that regulate major cellular functions such as the cell cycle, morphogenesis, and migration (Wang et al., 1993). Indeed, the bio-mechanical interactions between intracellular biological machinery and the surrounding microenvironment, through mechanosensitive receptors, create a normal physiological condition. However, in the setting of cancer, genetic and epigenetic (Burdziak et al., 2023), including biochemical and physical, perturbations in intracellular or extracellular environments, disrupt the cellular homeostasis leading to dysplasia and in most cases to the formation of a solid tumor. During tumor growth, the aforementioned normal forces are disrupted as a result of mechanical stresses originated from aberrant homeostasis, excessive growth and tissue dysplasia (Jain et al., 2014). Tumor growth is known to generate compressional forces that perturb the interstitial space, the ECM and the flow in the vasculature. In turn, perturbations in the interstitial space may cause accumulation of growth factors and cytokines that facilitate tumor growth; whereas disruption in the mechanical properties of ECM and flow may alter cellular behaviour (Malandrino et al., 2018b).

It is now evident that the mechanical aspects such as ECM stiffness are critical in regulating a wide range of cellular behaviour. For example, in the context of stem cell differentiation, human mesenchymal stem cells preferentially differentiate into neurons or osteocytes when cultured on substrates with stiffnesses matching brain or bone tissues respectively (Saha et al., 2008). In the setting of cancer transformation, when epithelial cells are cultured on a compliant substrate, normal cells show a decrease in the rate of DNA synthesis and an increase in the rate of apoptosis while transformed cancer cells maintain their growth and apoptotic characteristics (Wang et al., 2000). Furthermore, transformed cells exert higher traction forces compared to non-transformed cells. Consequently, the increase in ECM stiffness and the extent of compression can lead to activation and increased expression of Rho GTPase and downstream effectors as well as high levels of extracellular signal-regulated kinases (ERK) activity that facilitate the process of epithelial-mesenchymal transformation (EMT) (Klein et al., 2009).

## The impact of physical interactions in malignancy and invasion

Biochemical and biophysical characteristics of the ECM influences cell migration (Barenholz-Cohen et al., 2020) through variations in growth factors or chemokines (chemotaxis), stiffness (durotaxis), ligand density (haptotaxis), and topographical organization (contact guidance) to direct cells to target destinations (Wang et al., 2019). Recent advances in intravital imaging have revealed that cells can adopt a diverse set of migration strategies involving migration as single cells or collective strands, transitions between mesenchymal, epithelial, and amoeboid migration modes, deformation of the cell body and nucleus to squeeze through matrix pores, and remodeling of matrix structure to bypass the physical barriers presented by the ECM (Wang et al., 2019) (Figure 1B). Furthermore, heterogeneity of stiffness in tumor microenvironment, triggered by matrix remodeling can mechanically guide the tumor cells directional migration (Zhang et al., 2020).

EMT is a critical process in metastasis and involves loss of epithelial characteristics (Bocci et al., 2019), resulting from downregulation of cell-cell adhesion strength (for example, through loss of E-cadherin and cytokeratin) and acquisition of a mesenchymal phenotype via activation of migratory processes (for example, through upregulation of vimentin and N-cadherin). Taken together, the EMT process disrupts cellular force balances and polarity leading to morphological changes and detachment of tumor cells from the tumor epithelium (Thiery and Sleeman, 2006; Kalluri and Weinberg, 2009; Chaffer and Weinberg, 2011). By developing a high-throughput screening assay to track displacements generated by 3D cultured multicellular clusters, Leggett et al. (2020) showed a successive reduction in protrusive and circumferential tractions during EMT. Subsequently, the modulation of cellular shape and forces in combination with mechanisms favouring migration including proteolytic (matrix metalloproteinase), adhesive, protrusive (invadopodia) and contractile processes, promote invasion of cancer cells (Wolf and Friedl, 2009). Therefore, to facilitate their three-dimensional motility, cancer cells navigate through the ECM via invadopodial protrusions, balance cell-ECM adhesion, and apply contractile forces to squeeze through ECM pores and ultimately digest and remodel the ECM via force application and matrix metalloproteinase secretion.

The generation of a new tumor-specific vasculature that aids tumor growth, is concomitant with tumor development and transformation to malignancy and facilitates the escape of tumor cells into the circulation. In addition to biochemical signals (Chen et al., 2021), physical factors such as mechanical, hydrodynamical, and collective processes (Rieger and Welter, 2015) influence the generation and architecture of tumor-specific vasculature. Indeed, the growth of an avascular tumor is limited to a critical size (<1 mm) because of the inability of diffusion mechanisms to supply oxygen and soluble factors into the tumor core. This phenomenon results in the development of a necrotic/hypoxic region at the tumor core, which is surrounded by a highly proliferative outer rim. Therefore, robust vascularization mechanisms are recruited to boost tumor growth in order to enhance delivery of different factors, such as oxygen. Vascularization of the tumor and surrounding areas is initiated and maintained through the recruitment and activation of endothelial cells, mainly triggered by hypoxic pathways (Madsen et al., 2015), and signaling with other supporting cells in the tumor microenvironment. These include, immune cells, mesenchymal stromal cells and fibroblasts (Figure 1A) (Weis and Cheresh, 2011). The newly developed vessels perturb the normal architecture of blood and lymphatic networks and induce an aberrant interaction between the fluid and solid phases within the tumor leading to high levels of the interstitial fluid pressure and the lack of gas and nutrients (Figure 1A) (Koumoutsakos et al., 2013). Due to such a chemically and mechanically disordered tumor environment, direct drug delivery to solid tumors is often inefficient (Minchinton and Tannock, 2006). Following tumor vascularization, the combination of protracted tumor cell proliferation, continuous genetic transformations, angiogenesis and activation of bio-mechanical signaling pathways promote malignancy, invasion, and metastasis (Polyak and Weinberg, 2009; Wirtz et al., 2011; Mierke, 2019; Yang et al., 2020; Dou et al., 2022).

## Biomechanics of cancer cell during intravasation, circulation and extravasation

### Intravasation and extravasation

Intravasation describes the process by which individual or multiple tumor cells migrate away from the primary tumor site, cross the endothelial barrier to gain entry into the circulation (Figure 1C). Similarly, extravasation is the sequences of events where CTCs exit the bloodstream and invade the parenchyma of a secondary metastatic site. The efficiency of these events may be modulated by the external physical microenvironment that drives intracellular signaling, as well as the cell’s ability to perturb its inherent mechanical properties.

### External environment

To enter or leave the circulation, tumor cells must migrate across dense parenchymal tissue, which is composed of a network of highly cross-linked extracellular matrix and stromal cells. On average, pore sizes are on the scale of nanometers (<1 micron) (Laudani et al., 2020) while tumor cell size ranges 5–30 microns in diameter, suggesting that both matrix alterations and extreme cell deformation are required. One of the rate limiting steps of migration is the deformation of the tumor cell nucleus, which is approximately 5–10 times stiffer than the cytoplasm (Lammerding, 2011). As such, the matrix pore size and the deformability of the interstitial spaces are the factors that might dictate migration. Tumor cells are known to secrete matrix metalloproteinase (MMPs) like MMP2 to enable collagen proteolysis (Leong et al., 2014; Deryugina and Quigley, 2015; Micalet et al., 2022) and are shown to localize MT1-MMP at the leading edge of protrusion during migration, indicating an active role of degradation. Notably, the majority of vasculature is surrounded by a dense network of basement membrane (BM) proteins such as collagen IV and laminin, requiring further degradation processes like the secretion of gelatinases (e.g., MMP-9) prior to entering circulation (Maity et al., 2011; Sounni et al., 2011). Sikic et al. (2022) by tracking force-induced displacements and measuring local viscoelastic properties of Matrigel via magnetic micro-rheology, quantified tumor cell generated forces during invasion towards basement membrane in a 3D culture environment. They showed that protrusions extension involves stepwise increases in forces ranging from piconewtons to nanonewtons being exerted every few minutes. While matrix degradation could decrease the burden for the cell to undergo severe deformation, recent work has also shown the ability of tumor cells to exhibit substantial morphological changes in the absence of matrix loss (Wolf et al., 2003; Voura et al., 2013). This may involve large deformations of the tumor cell nucleus, which depends on mechanical properties of the nuclear lamina and organization of chromatin (Cao et al., 2016). In this regard, it is known that linker proteins (such as SUN domain-containing proteins) between nuclear and cytoplasmic (LINC) complexes can facilitate the proper positioning of the nucleus relative to the cell body to allow motility through narrow constrictions (Kraning-Rush et al., 2012; Denais et al., 2016).

The endothelium presents yet another barrier for tumor cell migration. Microvessels are lined with a single layer of endothelial cells connected to each other through junctional proteins such as VE-cadherin and CD31 (PECAM 1) that are responsible for the tight regulation of soluble factor transport between the blood and the surrounding tissue. The open gaps between endothelial cells are typically less than a few microns (McEvoy et al., 2022), suggesting that the transmigration of tumor cells may involve deformation of both tumor and endothelial cells. It has been shown that tumor cells can secrete inflammatory factors such as TNF-α which mediate endothelial junctional permeability and create discontinuities in the barrier to facilitate transmigration (Zervantonakis et al., 2012). Furthermore, the ability of tumor cells to anchor onto the endothelium through tumor-EC adhesion proteins such as integrins is critical for the generation of forces that allow translocation. Since integrins provide a connection between the ECM and the actin cytoskeleton, this mechanosensitive protein coupling and activation may lead to downstream intracellular signaling, thus determining the extent of intracellular forces required to maintain or obtain a certain cellular morphology. Also, BM mechanics at the primary and secondary tumour site plays a critical role in cancer progression, independent of tumour-mediated alterations; Reuten et al. showed that the BM stiffness is regulated through Netrin-4 in a laminin-binding-dependent manner by diluting laminin ternary node complexes. The more Netrin-4 molecules are present, the softer the laminin network and the more resistant it is to metastases formation (Reuten et al., 2021).

### Influence of extracellular physical signals on intracellular environment

Interestingly, it has been found that cancer cells are consistently softer than their non-cancerous counterparts (Rianna et al., 2020), and that the softening correlates positively with metastatic potential (Xu et al., 2012). The softer cytoplasm of more aggressive tumor cell lines is often correlated with a loss of cytoskeletal organization. Since the ability to migrate through dense matrix and endothelial barriers likely depends on the intrinsic mechanical properties of the cells, an increase in tumor cell compliance may act in its favor. Additionally, external stimuli including the presence of interstitial flow, ECM stiffness, 2D or 3D dimensionality (Galarza et al., 2020), and availability of binding sites for cell surface receptors (Wei et al., 2015) may influence the nature and deformability of the cell membrane and cytoskeleton. For instance, features that appear to be important for 2D motility—focal adhesion, stress fibres, broad lamellipodia—are largely absent for models of 3D invasion, particularly in invasive cancer cells (Shibue and Weinberg, 2009; Mierke, 2013). On the other hand, several mechanosensory proteins, such as vinculin, play an important role in tumor cell migration within reconstituted 3D matrices, but not in 2D motility (e.g., on plastic culture dishes coated with the same ECM proteins).

### Circulating tumor cells and shear stress

After entering the circulation, tumor cells are exposed to a variety of hemodynamic forces of flowing blood and collision with other cell types (Marrella et al., 2021). For example, shear flow can influence the motility of tumor cells and determine the likelihood that intercellular adhesions with the endothelium or circulating immune cells can occur. The average shear stresses a CTC experiences is estimated to be around 1–4 dyn/cm^2^ in the venous circulation, and 4–30 dyn/cm^2^ in arterial circulation. This is comparable to the levels of shear stress that cartilage and bone cells are subjected to on a daily basis from normal interstitial fluid movement (∼30 dyn/cm^2^), and to the level of renal epithelial cells undergo during hypertension (∼1 dyne/cm^2^) (Nagrath et al., 2007; Aceto et al., 2015; Au et al., 2016). Thus, it is highly possible that shear stress levels experienced by CTCs are significant enough to induce mechanotransductive cellular responses.

Additionally, shear flow can induce the deformation of tumor cells and influence their viability. For instance, CTCs migration in groups exhibit higher survival rates due to protection from deleterious shear stresses (Au et al., 2016). Thus, only tumor cells that overcome the effects of fluid shear stress and escape immunosurveillance can adhere to the vasculature and enter the tissues of the secondary site.

Tumor cell extravasation is thought to first require the firm adhesion and arrest of tumor cells on the endothelium. There are two mechanisms of tumor cell arrest (Craene and Berx, 2013): physical occlusion in capillaries narrower than the diameters of the CTC, and (Van Zijl et al., 2011) active adhesion between endothelial-tumor cell ligands/receptors. *In vivo*, tumor cells have mostly been observed to arrest in small capillaries of the brain and lung, suggesting the possibility of physical occlusion. Integrins and selectins are critical to determine tumor cell retention in several organs such as the lung and the liver, indicating that active cell adhesion may be involved in addition to pure physical occlusion. The increased ability of tumor cells to arrest on the endothelium may offer higher probability of exiting the bloodstream.

Adhesion to blood vessels is followed by cancer cell transendothelial migration (TEM). Depending on the vascular bed and tumor cell types, two mechanistically different routes are possible *in vivo*: transcellular (migration of CTCs through the EC body) and paracellular (moving between ECs junctions) (Herman et al., 2019). The latter is the most frequent accessed way for cancer cells to penetrate the vascular wall *in vitro*. This process involves many chemokines, receptors and intracellular signaling molecules leading to significant cytoskeletal changes of endothelial and cancer cells (Reymond et al., 2013). Additionally, Javanmardi et al. showed that mechanical properties of ECM, such as stiffness and porosity, regulate cell generated forces through mediating RhoA activity (Javanmardi et al., 2023). Furthermore, complex push–pull forces generated by cancer cell actin-rich protrusions are essential to initiate and drive transendothelial migration (Javanmardi et al., 2023).

Interactions with other cells in the blood have also been shown to be critical. For example, adhesion to platelets through tumor integrin αvβ3 can promote tumor-platelet aggregation, which leads to the protection of tumor cells from shear flow. Additionally, platelets can secrete pro-extravasation factors such as platelet-derived nucleotides, which act to increase endothelial permeability and facilitate the transmigration of tumor cells (Labelle et al., 2011; Schumacher et al., 2013). Interactions with circulating neutrophils can increase tumor cell retention and extravasation via neutrophil CD11b, endothelial ICAM-1; and the success of these interactions decline with increasing shear rates resulting from blood flow (Peng et al., 2007; Huh et al., 2010).

## Probing cancer biomechanics and emerging technologies

Recent technological advances provide a better insight on the biomechanical phenomena during cancer cell dissemination by probing morphological changes, mechanical properties and force interactions between cells and the extracellular environment. While advanced light microscopy techniques such as optical super-resolution imaging offer unprecedented information about the nano-scale molecular organisation of the cell (Colin-York et al., 2019a) and its link to cellular morphology and function, they are mostly limited to isolated two-dimensional (2D) cultures in which a monolayer of cells are grown on flat plastic or glass substrates. To access high resolution tumor pathophysiology *in vivo*, intravital imaging has revealed some fascinating morphological changes and cell migration processes associated with invasion and intravasation (Jain et al., 2002). Three dimensional (3D) interactions among cells and the extracellular environment are unique at all stages of metastasis and cannot be recapitulated in conventional 2D cultures. On the other hand, under *in vivo* conditions, it is extremely difficult to fully follow the temporal evolution of 3D interactions and run parametric studies to dissect the role of different factors. Therefore, advanced 3D engineered models such as microfluidic based approaches, have been used in recapitulating key biomechanical features that are specific to each step of metastasis (Chen et al., 2017; Agrawal et al., 2022; Straehla et al., 2022; Watson et al., 2022) and to quantify tumor cell secretions at the single cell resolution over a long period of culture (Hassanzadeh-Barforoushi et al., 2020). Together, they have allowed the investigation of a variety of cellular events with high spatiotemporal resolution and under tunable environments (Malandrino et al., 2018a).

**FIGURE 2:**
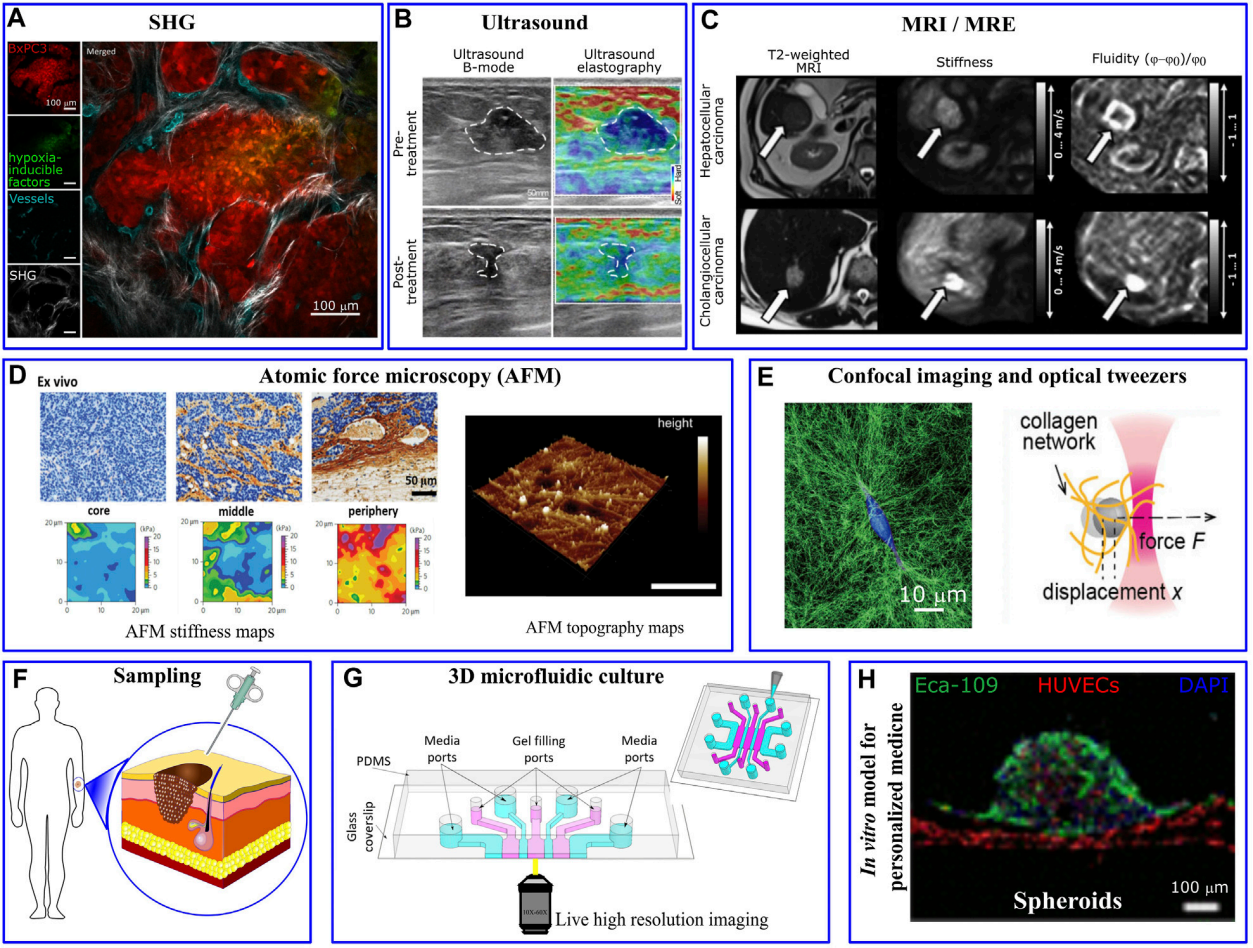
Mechanical/structural properties of the tumor microenvironment and microfluidic platform in personalized medicine. **(A)** Different tumor associated collagen signatures have been captured via *in vivo* second harmonic generation (SHG) imaging of mouse pancreatic tumor. Irregularly shaped and newly formed vessels are observed in the inner regions of the tumor, taken from Samuel et al. (2023). **(B)** Ultrasound and **(C)** Magnetic resonance elastography show various levels of tumor stiffness that can inform the planning of resection procedures, taken from Liu et al. (2023) and Sauer et al. (2023), respectively. **(D)** High resolution AFM stiffness maps of mouse mammary tumor shows highly heterogeneous tumor mechanical properties with a soft signature at the tumor core and significantly stiffer periphery. Taken from Plodinec et al. (2012). AFM topographic maps provides helpful information about matrix architecture (taken from Budden et al. (2021)). **(E)** Single MDA-MB-231 cancer cell embedded within collagen-I gel significantly deformed and remodeled collagen fibres imaged via confocal reflection microscopy and changed the gel stiffness quantified via optical tweezers. Taken from Han et al. (2018). **(F)** Cells that are derived directly from a patient are usually scarce. **(G)** The few isolated cells can be 3D-cultured in a biomimetic microfluidics device for drug discovery, personalized drug screening, or to investigate the impact of biomechanical/biochemical factors on cellular behaviour (taken from Whisler et al. (2023)). **(H)** Perfusable vascularized tumor spheroid-on-a-chip model incorporates patient’s isolated cells as a personalized medicine approach (taken from Hu et al. (2022)).

The measurement of mechanical properties is critically important in cancer biomechanics. Mechanical changes and alternations in the composition, architecture and stiffness of tumor microenvironment regulate tumor growth, transformation to malignancy, and invasion (Pickup et al., 2014) being therefore critical aspects in cancer progression (Cox and Erler, 2011; Lu et al., 2012; Popova and Jücker, 2022). For instance, tumor-associated collagen exhibits specific features due to variation in fiber orientation and collagen deposition. Notably, three distinct tumor-associated collagen signatures (TACS) have been identified in relation to human breast cancers: the accumulation of collagen fibers around small tumors (TACS-1), the straighten fibers in the vicinity of non-invasive tumors (TACS-2) and the perpendicular alignment of collagen fibers at the tumor periphery (TACS-3) (Warli et al., 2023). Such irregularities have been visualized through microscopy techniques, such as second harmonic generation, which highlights the structural transformations in collagen fibers (Figure 2A). Furthermore, aberrant mechanical properties of the tumor environment can be measured as a diagnostic tool (Plodinec et al., 2012). The mechanical properties of the tumor *per se*, as well as the micro-mechanical features of tumor environment have been characterized through a number of *in vivo*, *ex vivo*, and *in vitro* based assays at micro-nano- scale resolutions. As an alternative to conventional palpation methods, *in vivo* elastography measurements, such as ultrasonography (Wells and Liang, 2011), optical coherence tomography (Kennedy et al., 2015), and magnetic resonance imaging (Venkatesh et al., 2008), revealed significant stiffening of tumor tissues particularly in malignant tumors (Ramião et al., 2016; Ishihara and Haga, 2022). For example, pancreatic cancer tissue exhibits greater stiffness of ∼6 kPa compared to the 1–3 kPa range observed in normal pancreatic tissue (Itoh et al., 2016). Lung solid tumors register stiffness levels of ∼20–30 kPa, whereas normal lung parenchyma stiffness typically ranges from 0.5 to 5 kPa (Miyazawa et al., 2018). For mammary tissues, cancerous tissue displays a significantly higher stiffness of around 4 kPa, in stark contrast to the ∼0.2 kPa stiffness found in normal mammary tissue (Paszek et al., 2005). Elastography-based ultrasound techniques offer a non-invasive and real-time approach to assessing tissue stiffness, enhancing the diagnostic accuracy, and providing valuable information for personalized cancer treatment (Figure 2B). Interestingly, Golatta et al. showed that adding combined shear wave elastography and strain elastography to routine B-mode breast ultrasound could help reduce the number of unnecessary biopsies in breast diagnostics by ∼35% (Golatta et al., 2022). However, increased stiffness signature, that has been a well-known characteristic of solid tumors, alone has a limited prognostic power. The prognostic potential enhances when examining other rheological properties of the tumor microenvironment through the application of *in vivo* multifrequency magnetic resonance elastography (MRE). Employing this technique, Sauer et al. (2023) outlined a roadmap for prognosis of a tumor’s aggressiveness and metastatic potential based on stiffness, fluidity, spatial heterogeneity, and texture of the tumor (Figure 2C). They showed that cancer progression is accompanied by tissue fluidization, where portions of the tissue can change position across different length scales.”

Alterations in cellular and extracellular composition/structures contribute to the increased tumor stiffness. For example, excessive proliferation of cancer cells and activation of stromal cells such as cancer associated fibroblasts (CAFs) (Calvo et al., 2013), induce ECM remodeling (Bertero et al., 2019); whereas tumor growth induces solid stress within the tumor itself (Nia et al., 2017; Nia et al., 2020). The presence of growth-induced solid stresses in tumors had been under suspicion for some time, as these stresses were largely estimated using mathematical models (Stylianopoulos et al., 2012). However, in the past decade various experimental techniques have emerged to directly measure such stresses. For instance, Campàs et al. (2014) introduced incompressible oil microdroplets into 3D cell aggregates and live embryonic tissue to assess the local anisotropic forces. Dolega et al. (2017) and Lee et al. (2019) employed compressible polyacrylamide microdroplets to quantify both radial and circumferential components of solid stress within spheroids. Additionally, L et al. (2017) showed that CAFs promote tumor invasion and metastasis through exerting mechanical forces on cancer cells which are mediated via adhesion proteins involving N-cadherin at the CAF membrane and E-cadherin at the cancer cell membrane. On the other hand, tumor vascularization leads to aberrant interactions between the blood flow that infiltrates and surrounds the tumor and the increased interstitial fluid pressure (Jain et al., 2014). To dissect the contribution of the different components of tumor microenvironment, high-resolution mechanical measurement techniques, such as AFM (atomic force microscopy), have been widely applied. AFM nanomechanical indentation tests on tumor slices showed a soft mechanical signature within the tumor core, where cancer cells are abundant, while the adjacent peripheral regions are stiffened mostly due to collagen alignment (Figure 2D) (Laklai et al., 2016; Stylianou and Stylianopoulos, 2016).

At the single cell level, several experimental methods such as magnetic twisting cytometry, magnetic and optical tweezers (Figure 2E) and AFM have been utilized to perturb small regions of the cell and characterize mechanical properties, such as stiffness, of isolated cancer cells. Furthermore, Kim et al. (2018) developed a multi-parametric single-cell-analysis method in which different cell lines were transported through a microfluidics channel to measure their mechanical properties. By defining a whole-cell deformability index, they showed that malignant and non-malignant cell lines have different mechanical signatures. Traction Force Microscopy has been widely utilized to observe force interaction of cells and measure cell-generated forces (Colin-York et al., 2019b; Javanmardi et al., 2021; Li et al., 2021). This force probing technique is based on imaging the cell-induced displacement of fiducial markers, embedded/targeted within extracellular environment, and use the computational procedure to back-calculate the cellular forces that generated the displacements. To streamline and simplify the computation procedures involved in TFM, Barrasa-Fano et al. (2021) developed TFMLAB, a MATLAB software package for 4D TFM. Interestingly, while highly metastatic cancer cells exhibited a softer phenotype (∼0.5 kPa) compared to non-metastatic cells (∼2 kPa), they generate stronger forces (Kraning-Rush et al., 2012; Kristal-Muscal et al., 2013) (∼300 nN for MDA-MB231 vs ∼150 nN for MCF10A) (Cross et al., 2007; Gal and Weihs, 2012)”, allowing them to squeeze through 3D ECM and metastasize more readily (Coughlin et al., 2013). Also, in the cluster level, contractile forces generated by tumor spheroid have been measured and normalized in a scale-independent manner (Mark et al., 2020).

The mechanical behavior of ECM, cancer cells, and tissues are often assumed to be elastic solids for simplicity and their time-dependent mechanical responses are frequently overlooked. However, it has been demonstrated that ECM exhibits a more complex mechanical behavior, including viscoelasticity, mechanical plasticity, and nonlinear elasticity (Chaudhuri et al., 2020) and indeed, the viscoelasticity of ECM plays a fundamental role in the progression of cancer (Mierke, 2021). Additionally, cellular behavior is not affected by only the mechanics of solid compartment of the ECM; viscosity of the extracellular fluid has also been shown to facilitate tumor cell migration and dissemination on 2D surfaces and in 3D spheroids (Bera et al., 2022). In addition to ECM, cancer cells themselves typically show a lower levels of viscosity and membrane tension compared to healthy cells (Ren et al., 2023). Moreover, cytoplasm of the living cells also has been shown to behave as a poroelastic material (Moeendarbary et al., 2013) with an enhanced diffusion coefficient for cancer cells (Ren et al., 2023). At the tissue level, higher levels of viscos (loss) modulus in cancerous tissues (Deptuła et al., 2020) has improved the diagnostic accuracy and capabilities in ultrasound (Nabavizadeh et al., 2019) and MRE techniques (Reiter et al., 2022).

## Mechanobiology and challenges with translation

### Modulation of physical properties as a path of pharmacological intervention

It is now accepted that the ability of a cancer cell to successfully invade the surrounding ECM and cross endothelial barriers during metastasis requires a finely regulated set of mechanical properties. Thus, any alterations to either the surrounding physical environment or the cell cytoskeletal organization could potentially become a target of therapeutic intervention.

For example, neutralization of matrix degrading MMPs may increase steric hindrance and decrease the ability of tumor cells to cross ECM during the invasion phase. While some tumor cells appear to be resistant to these perturbations, being able to migrate in an amoeboid manner (without degradation), inhibition of the matrix-degradation may prove to be effective in other types of cancers. Nuclear deformation is also seen to be a rate-limiting factor in migration through narrow constrictions (Cao et al., 2016). Thus, overexpression of proteins such as lamins that maintain nuclear stiffness, could hinder large nuclear deformations and delay the rate at which tumor cells invade (Wirtz et al., 2011; Díaz de la Loza et al., 2017).

Once tumor cells have spread into the circulation, it might still be possible to intervene at the stage of intravascular adhesion and arrest. For instance, perturbation of tumor-endothelial adhesion molecules such as E-selectin, CD44, PODXL, VCAM1 or ICAM1 could decrease the rate of heterotypic interaction under shear flow, resulting in lower tumor cell retention rates in the circulation and limitation of metastatic seeding at the distant site (Rosette et al., 2005; Zen et al., 2008). Similarly, targeting tumor-interacting adhesion proteins on immune cells like platelets and leukocytes could yield comparable anti-metastatic effects. Care, however, must be taken in all cases to minimize the perturbation to the normal homeostatic functions of the non-cancer cells involved.

It is still a matter of debate whether the physical characteristics of cancer cells, such as deformability and stiffness, are conserved through generations, or whether these are developed in response to heterogeneous extracellular mechanical and biochemical cues, spread over time and space. Whether these physical properties are inherited or acquired throughout different stages of metastasis, it might be possible to alter them, either through pharmacological inhibition or through the activation of proteins affecting cell mechanics. Together, it may be possible to exogenously achieve a set of optimal mechanical microenvironmental conditions (i.e., cell stiffness, matrix density and pore size, interstitial fluid forces) such that the likelihood of proceeding through the metastatic cascade is lowered.

Lastly, applicability of targeting mechanics is a relatively new approach compared to genes or molecular biomarkers methods and therefore most of the clinical interventions leading to mechano-therapeutics are still in the trial phase. Jain et al. (2014), Huang et al. (2021), Jiang et al. (2022), and Di et al. (2023) compiled a list of such drugs; among which it is worth mentioning: Cilengitide, a selective αvβ3/αvβ5 integrin inhibitor that has been assessed in phase III clinical trial for treating glioblastoma or GB 2064 (formerly PAT-1251) and a LOXL2 inhibitor that lowers collagen accumulation and ECM stiffness and currently being evaluated in phase II clinical trial for treating Myelofibrosis. Additionally, pamrevlumab is being tested in phase III in patients with locally advanced, unresectable pancreatic cancer. Pamrevlumab is a monoclonal antibody that targets connective tissue growth factor, thereby reducing the fibrotic tissue and making unresectable tumors amenable to surgical excision (Sheridan, 2019).

## Mechanobiology in personalized medicine

It is well known that tumor cells can vary widely according to the oncogenic background. Often, further heterogeneity occurs within a metastatic tumor when different patients are considered. Lab-on-a-chip systems are particularly well poised to address such questions related to patient -specificity with key advantages such as the requirement of low sample and reagent inputs, which are often scarce when derived directly from a patient (Figure 2F). Further, the ability to multiplex and perform high-throughput experiments is particularly amenable in lab-on-a-chip assays, enabling clinical level drug screening to be done on a person-to-person basis. For instance, microfluidic assays that recapitulate the surrounding mechanical environment of tumor cells (matrix stiffness, composition, pore size) can be used to understand the migratory phenotype of individual tumor cells as a typical indicator of potential malignancy, and how different pharmacological perturbations modulate this behavior (Pathak and Kumar, 2012; Polacheck et al., 2013; Polacheck et al., 2014) (Figure 2G). More complex *in vitro* models that mirror not only the surrounding ECM but also other physical cues (such as vasculature and stromal cells and fluid flows (Achilli et al., 2012; Kim et al., 2015; Whelan et al., 2023) can be employed in a multiplexed and high throughput manner to understand how these variables influence tumor cell migration in a patient-specific manner. This is particularly important as the recent FDA modernization act aims to integrate complex *in vitro* models of different diseases into the drug development process. For instance, Prolyl hydroxylases (PHDs) inhibitors have been shown to improve drug distribution in mice tumors and increase the effectiveness of chemotherapy. To demonstrate such effects in human cell models, Hu et al. (2022) employed a perfusable vascularized spheroid-on-a-chip model to simulate tumor microenvironment *in vivo*. They showed that dimethylallyl glycine improves the efficacy of the anticancer drugs paclitaxel and cisplatin in human esophageal carcinoma (Eca-109) spheroids (Figure 2F). Additionally, this could allow further understanding of the effects of external physical features such as matrix composition, stiffness, pore size, tumor cell adhesion molecules, and MMPs, on tumor migratory abilities and potentially tumor type characterization.

In addition to personalized drug screening under relevant biomechanical condition, it may also be possible phenotyping tumor cells and assess for instance their degree of malignancy through mechanical measurements on patient derived single tumour cells. Stiffness values of tumor cells or even aggregates of cells like tumor spheroids can now be measured using various contact-based (e.g., AFM) and non-contact-based methods (e.g., Brillouin imaging (Scarcelli et al., 2015; Roberts et al., 2021)); correlations can be made between these stiffness values and the invasive properties of these cells, through parallel *in vitro* screening experiments, and even using clinical tumor phenotyping data. This could allow extrapolating the metastatic potential from a small number of patient-derived samples and would prove to be a useful predictor when used in conjunction with traditional diagnostic methods.

Lastly, a deep understanding of the physical and structural properties of the surrounding tumor matrix might be useful for optimizing drug delivery to tumor sites. As an example, second harmonic imaging is now widely used to quantify the structural composition of collagens in the tumor microenvironment (Condeelis and Weissleder, 2010), and allow the characterization of pore size and fiber thickness, which may have a large influence on the effectiveness of drug transport. Obtaining this data for patient-specific tumors and subsequent computational modelling of drug transport based on these parameters may aid in the construction of a more optimized drug delivery system in the clinic.

## Discussion

Accumulated evidence has demonstrated the fundamental role of mechanobiology in cancer research as the physical properties of both cells and their microenvironment play a crucial role in the development and progression of cancer. In this short review we summarized the role of cellular biomechanical properties, e.g., stiffness, adhesion and motility, morphology, deformability, and contractility, as well as microenvironment properties such as ECM stiffness, tissue architecture, blood vessel permeability, and interstitial flow, in different steps of the tumor dissemination. Indeed, mechanobiological insight could benefit researchers and clinicians in various ways; For instance, mechanical quantification at the tissue level has been used as a diagnosis or prognosis tool for various types of cancer. Advancements in the *in vivo* non-invasive techniques such as MRE and ultrasound elastography as well as *ex vivo* techniques such as AFM have improved our diagnosis abilities in the past decade. Furthermore, at the cellular level, mechanobiology can contribute significantly to our comprehension of molecular and genetic alterations in cancer. The interplay between mechanical forces and cellular behaviors can influence how genes are expressed, proteins are synthesized, and signaling pathways are activated. Such insights are of crucial importance in the development of new therapeutic strategies, as many ongoing clinical trials target components of the extracellular matrix (Lampi and Reinhart-King, 2018; Huang et al., 2021) and proteins associated with mechanosignaling pathways (Jiang et al., 2022). Nonetheless, owing to the diversity in oncogenic backgrounds and the inherent variability within metastatic tumors, distinct therapeutic strategies are required for individual patients; In this regard, microfluidics technology holds substantial promise in advancing personalized medicine through its ability to create controlled environment for studying individualized patient samples and responses. Nevertheless, there are intriguing avenues for further research to enhance the translatability of mechanobiological knowledge derived from these platforms into clinical practice. This includes integration of the cancer mechanobiology insight with the data from genomics, transcriptomics, and proteomics approaches; this, in combination with single cell data analysis (obtained from limited number of tumor cells harvested from patient’s blood or tissue) could augment our understanding of mechanotransducive pathways involved in cancer. Moreover, by introducing immune cells along with other supporting cell types into the 3D tumor-mimicking microenvironment established within microfluidic chips, we can investigate effects of mechanical signals on immune cell behavior, as well as their implications for immunotherapy responses and the emergence of resistance.
